# Glucagon-like peptide-1 protects against ischemic left ventricular dysfunction during hyperglycemia in patients with coronary artery disease and type 2 diabetes mellitus

**DOI:** 10.1186/s12933-015-0259-3

**Published:** 2015-08-08

**Authors:** Liam M McCormick, Patrick M Heck, Liam S Ring, Anna C Kydd, Sophie J Clarke, Stephen P Hoole, David P Dutka

**Affiliations:** Department of Cardiovascular Medicine, University of Cambridge, Cambridge, UK; Department of Cardiovascular Medicine, ACCI Level 6, Addenbrooke’s Hospital, Box 110, Hills Rd, Cambridge, CB2 0QQ UK

**Keywords:** Hyperglycemia, Coronary disease, Diabetes mellitus, Glucagon-like peptide, Stress echocardiography

## Abstract

**Background:**

Enhancement of myocardial glucose uptake may reduce fatty acid oxidation and improve tolerance to ischemia. Hyperglycemia, in association with hyperinsulinemia, stimulates this metabolic change but may have deleterious effects on left ventricular (LV) function. The incretin hormone, glucagon-like peptide-1 (GLP-1), also has favorable cardiovascular effects, and has emerged as an alternative method of altering myocardial substrate utilization. In patients with coronary artery disease (CAD), we investigated: (1) the effect of a hyperinsulinemic hyperglycemic clamp (HHC) on myocardial performance during dobutamine stress echocardiography (DSE), and (2) whether an infusion of GLP-1(7-36) at the time of HHC protects against ischemic LV dysfunction during DSE in patients with type 2 diabetes mellitus (T2DM).

**Methods:**

In study 1, twelve patients underwent two DSEs with tissue Doppler imaging (TDI)—one during the steady-state phase of a HHC. In study 2, ten patients with T2DM underwent two DSEs with TDI during the steady-state phase of a HHC. GLP-1(7-36) was infused intravenously at 1.2 pmol/kg/min during one of the scans. In both studies, global LV function was assessed by ejection fraction and mitral annular systolic velocity, and regional wall LV function was assessed using peak systolic velocity, strain and strain rate from 12 paired non-apical segments.

**Results:**

In study 1, the HHC (compared with control) increased glucose (13.0 ± 1.9 versus 4.8 ± 0.5 mmol/l, p < 0.0001) and insulin (1,212 ± 514 versus 114 ± 47 pmol/l, p = 0.01) concentrations, and reduced FFA levels (249 ± 175 versus 1,001 ± 333 μmol/l, p < 0.0001), but had no net effect on either global or regional LV function. In study 2, GLP-1 enhanced both global (ejection fraction, 77.5 ± 5.0 versus 71.3 ± 4.3%, p = 0.004) and regional (peak systolic strain −18.1 ± 6.6 versus −15.5 ± 5.4%, p < 0.0001) myocardial performance at peak stress and at 30 min recovery. These effects were predominantly driven by a reduction in contractile dysfunction in regions subject to demand ischemia.

**Conclusions:**

In patients with CAD, hyperinsulinemic hyperglycemia has a neutral effect on LV function during DSE. However, GLP-1 at the time of hyperglycemia improves myocardial tolerance to demand ischemia in patients with T2DM.

Trial Registration: http://www.isrctn.org. Unique identifier ISRCTN69686930

## Background

Hyperglycemia is common in patients presenting with acute coronary syndromes (ACS) and a powerful predictor of morbidity and mortality [1]. Although it has a number of direct detrimental effects on the ischemic myocardium [2], hyperglycemia is also representative of an acute metabolic stress characterised by high adrenergic-mediated levels of free fatty acids (FFA), insulin resistance and impaired glucose utilization [3]. Attempts to modulate these metabolic derangements with insulin-based strategies have failed to demonstrate consistent clinical benefits [4, 5] and are yet to be established in the management of patients with ACS [6, 7]. There is therefore an ongoing requirement for the development of new pharmacological therapies to address this problem.

Glucagon-like peptide-1 (GLP-1) is an incretin hormone which regulates glucose metabolism by stimulating insulin secretion, suppressing glucagon and promoting glucose uptake into muscle and adipose tissue. These effects are dependent on the prevailing glucose concentration so that the risk of hypoglycemia is minimized [8]. As a result, the need for intensive monitoring and concomitant glucose infusions associated with insulin-based metabolic strategies is obviated [9]. As well as these glucoregulatory effects, GLP-1 also has binding sites in the heart, and clinical studies demonstrating the cardioprotective properties of incretin-modulating agents are continuing to emerge [10–16]. GLP-1 therefore appears attractive as a potential therapeutic adjunct for metabolic manipulation in patients with ischaemic heart disease (IHD).

We have previously used a model of hyperinsulinemic euglycemic clamping (HEC) during dobutamine stress echocardiography (DSE) to demonstrate that alteration of the myocardial metabolic environment in patients with obstructive coronary artery disease (CAD) can attenuate left ventricular (LV) ischemic dysfunction, especially in those with insulin resistance [17]. However, the effects of hyperinsulinemic hyperglycemia (HH) in this setting have not been investigated and remain unclear. This study was therefore undertaken to answer two questions relating to patients with CAD. Firstly, we sought to investigate the effects of HH on LV performance during dobutamine stress. Secondly, we sought to investigate the effect of GLP-1 modulation on myocardial function in the setting of hyperglycemia in patients with type 2 diabetes mellitus (T2DM).

## Methods

### Study population

Patients with obstructive CAD (at least one proximal stenosis >70% in at least one epicardial coronary artery) and preserved resting LV systolic function were invited to participate. All patients had undergone recent coronary angiography before enrolment in the study. Exclusion criteria included LV ejection fraction (EF) <40% (either on echocardiography or LV angiography), regional wall motion abnormalities at rest, a history of previous myocardial infarction within the preceding 3 months, conduction abnormalities, valvular heart disease, patients taking insulin, dipeptidyl peptidase-4 (DPP4) inhibitors or GLP-1 receptor agonists, and those with permanent pacemakers. The study protocol was approved by the local ethics committee (REC number 08/H0304/68), and conformed with the guidelines set out in the Declaration of Helsinki. All participants gave written informed consent. The trial number was ISRCTN69686930

### Study design

Two separate studies were undertaken:

- *Study 1: effects of hyperglycemia on myocardial performance during dobutamine stress*

**Fig. 1:**
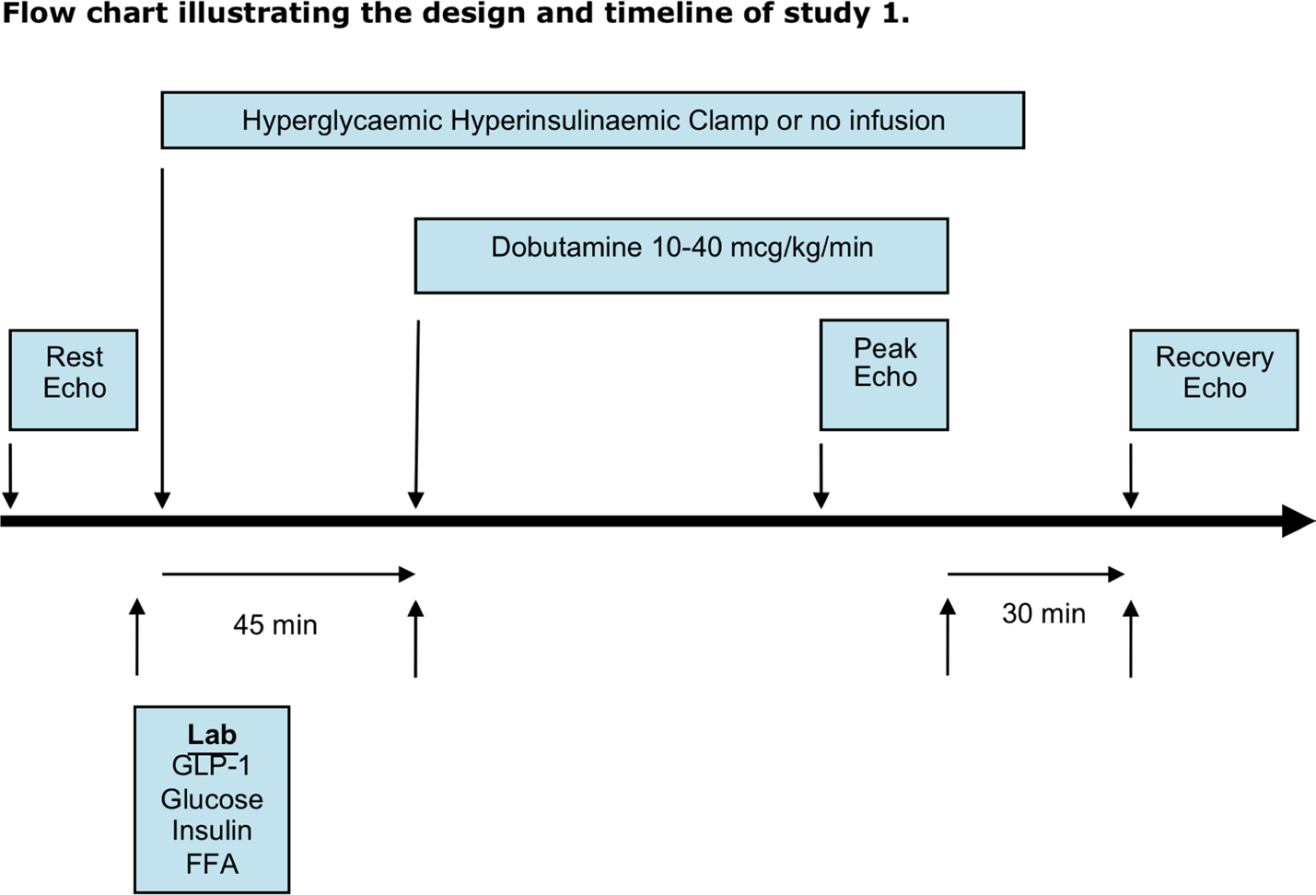
Flow chart illustrating the study design and timeline of study 1.

Each subject underwent two DSEs, performed approximately one week apart. One DSE, determined randomly, was performed during the steady-state phase of a hyperinsulinemic hyperglycemic clamp (HHC) and the other DSE acted as a control (Fig. 1).

- *Study 2: effects of GLP-1 (7-36) on myocardial performance during dobutamine stress in the setting of hyperglycemia*

**Fig. 2:**
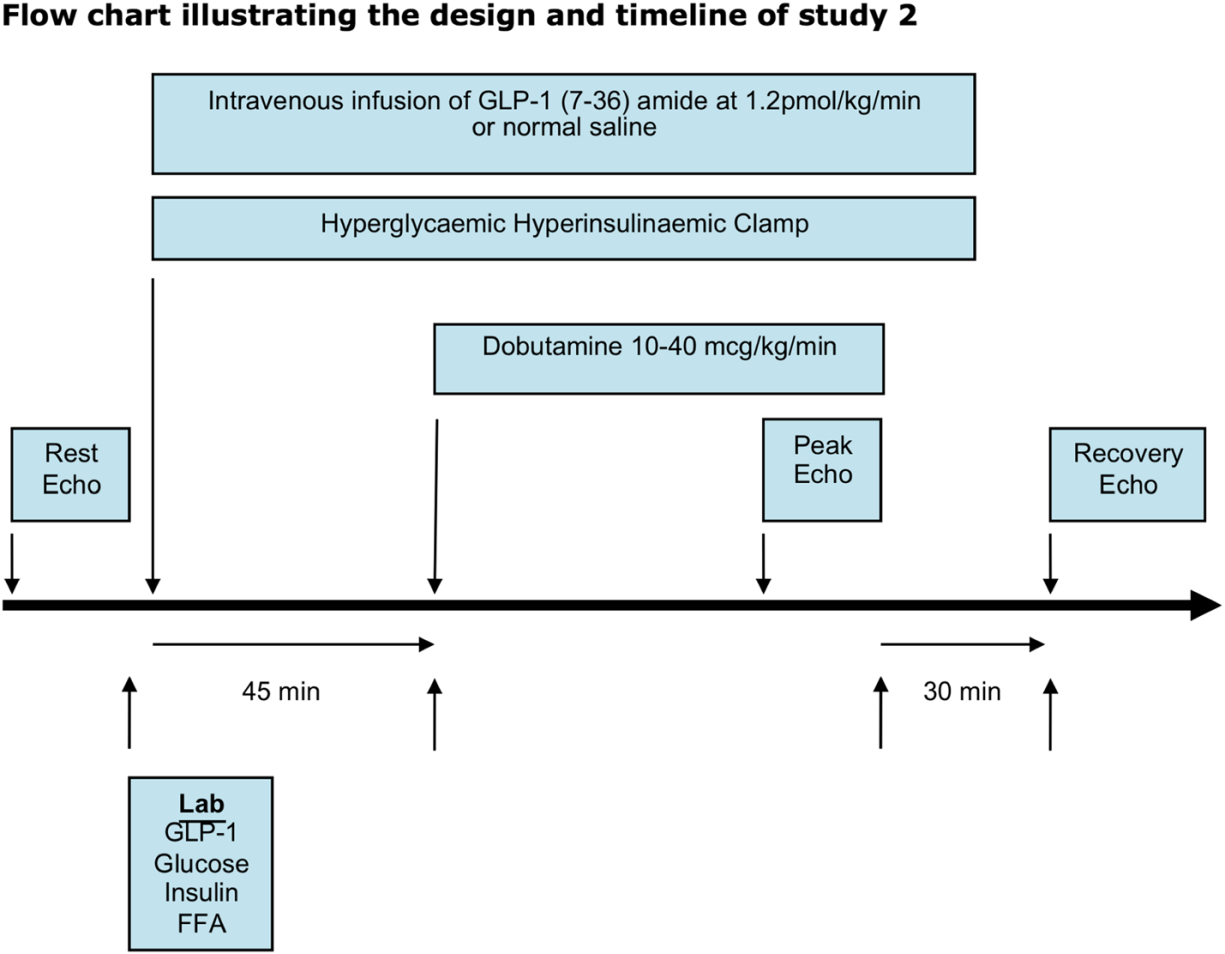
Flow chart illustrating the study design and timeline of study 2.

Consecutive patients with T2DM underwent DSE during the steady-state phase of a HHC on two separate occasions, approximately one week apart. One DSE, determined randomly, was performed during an intravenous infusion of GLP-1 (7-36) amide (Bachem, Germany) at a dose of 1.2 pmol/kg/min, and the other DSE (control HHC) during an intravenous infusion of normal saline (Fig. 2).

### Hyperinsulinemic, hyperglycemic clamp

The HHCs were performed as described previously [18]. After patients had fasted overnight, a cannula was inserted into a vein in the antecubital fossa for infusion of glucose. A second cannula was inserted in the opposite arm, which was arterialized using a heating pad set at 50°C. In order to raise the blood glucose concentration to approximately 13 mmol/L, a bolus injection of 0.3 mg/kg of 25% glucose was given over the first minute of the clamp. Once the bolus was administered, a maintenance infusion of 20% glucose was commenced at 5 mg/kg/min (study 1) or 2.5 mg/kg/min (study 2) and this rate was kept constant for the first 10 min. Blood glucose levels were sampled every 5 min from the opposite cannula and analyzed using the glucose oxidase method (YSI 2300, YSI Life Sciences, Ohio, USA). Thereafter, the glucose infusion rate was adjusted according to the plasma glucose concentration, aiming to maintain it at 13 mmol/L. The clamp was then continued for a minimum of 45 min. Once steady state was achieved, as indicated by three consecutive blood glucose values that were within 5% of one another, the subjects underwent DSE as described below. After cessation of dobutamine, the glucose infusion was continued for 15 min and then gradually reduced over the next 30 min to avoid rebound hypoglycemia.

### Dobutamine stress echocardiography

A standard clinical protocol for DSE was used. Subjects attended for both studies after an overnight fast and their beta-blocker was withheld for 48 h prior to their attendance. Oral hypoglycemic agents were omitted on the morning of the study. Dobutamine was administered intravenously via the antecubital fossa using an infusion pump in incremental doses (10 µg/kg/min initially, then increased at 3-min intervals to 20, 30 and 40 µg/kg/min if tolerated) and if necessary, up to 2 mg of atropine was given to achieve the target heart rate. Subjects underwent continuous 12-lead ECG monitoring, with blood pressure being recorded at baseline and at the end of each stage of dobutamine. Criteria for stopping the test were achievement of target heart rate of (220-age) × 0.85 bpm, ischemic ECG changes (>2 mm ST depression), angina, systolic blood pressure increase to >240 mmHg or decrease to <100 mmHg, and severe arrhythmias. Two-dimensional echocardiography (Vivid 7, GE Medical Systems) was performed with the patient in the left recumbent position, and images were recorded at rest, peak stress and in recovery. Three cardiac cycles of the apical 4-, 3-, and 2-chamber views were captured with tissue Doppler imaging (TDI). The image sector width was kept as narrow as possible to maximize the frame rate. All recordings were made in gently held mid-expiration to minimize beat-to-beat variability, and the data were stored for subsequent off-line analysis (EchoPac Version 11, GE Medical Systems).

In addition to blood glucose sampling, blood samples were taken in the fasted state to measure insulin, FFA, and GLP-1(7-36) concentrations at baseline, at the steady state stage of the HHC immediately prior to commencement of the DSE (pre-DSE), during peak dobutamine stress and in the recovery phase at 30 min after cessation of dobutamine. The syringes for the collection of GLP-1 samples were pre-prepared with DPP-4 inhibitor (Millipore) to prevent GLP-1 degradation. Plasma GLP-1 levels were measured using a commercially available assay (Meso Scale Discovery, Rockville, MD, USA).

### Echocardiographic analysis

**Fig. 3:**
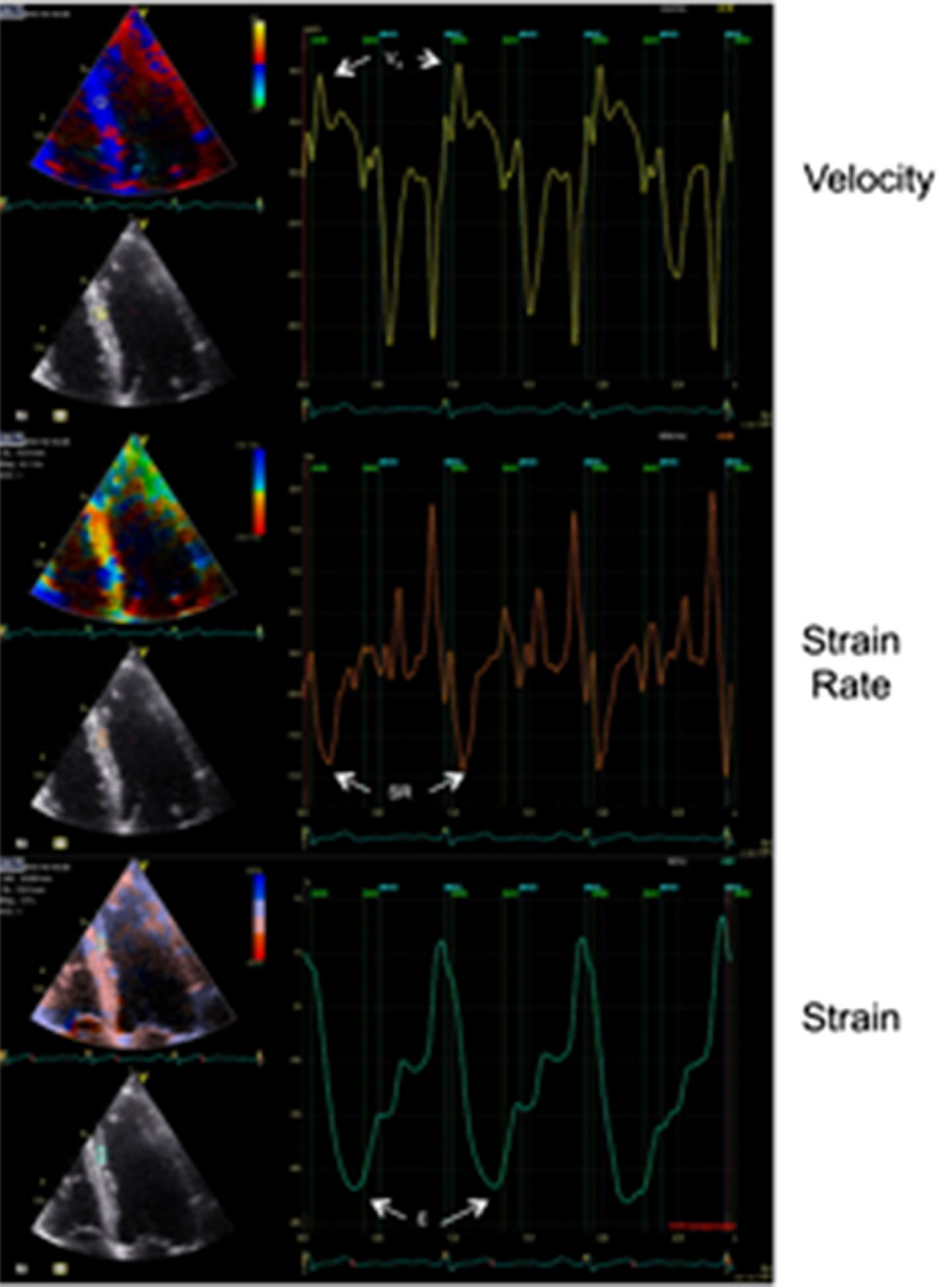
Myocardial velocity profile sampled from the mid inferoseptum with corresponding strain rate and strain curves.

The scans were analyzed off-line by a reviewer who was blinded to the treatment strategy. Regional wall LV motion was assessed using a 12-segment model comprising the base and mid level of six regional walls (anterior, anterolateral, anteroseptal, inferior, inferolateral, and inferoseptal) obtained from the three apical views. LV volumes and EF were calculated using the Simpson biplane method according to the guidelines of the American Society of Echocardiography. Global LV function was also assessed by mitral annular systolic velocity (MASV) averaged from six sites [19]. Peak systolic tissue velocity, and strain and strain rates were calculated from tissue Doppler velocity data averaged over three consecutive beats (Fig. 3). The timings of aortic valve opening and closure were made from the tissue Doppler waveform. The MYDISE study demonstrated that CAD could be diagnosed accurately and objectively from off-line measurements of myocardial velocities recorded by tissue Doppler echocardiography during dobutamine stress [20]. In particular, strain rate imaging has been shown to provide objective evidence of inducible ischemia [21], and may be a superior parameter to peak tissue velocity [22].

A diameter stenosis of >70% stenosis on coronary angiography was considered hemodynamically significant. Myocardial segments were assigned to the perfusion territories of stenosed vessels, considering the left anterior descending coronary artery to supply the anterior and anteroseptal segments, the right coronary artery (when dominant) to supply the inferior and inferoseptal segments, and the circumflex artery to supply the anterolateral and inferolateral segments.

### Statistics

The number of subjects had been calculated on the basis of previous work in our laboratory in patients with CAD, where EF increased from 70.8 ± 5.0% to 77.0 ± 4.4% when dobutamine stress was performed during an intravenous infusion of GLP-1 (7-36). To detect a change in global LVEF of 5% after dobutamine stress (standardized effect size of 1), 12 patients were required (paired *t* test, α = 0.05, β = 0.10). Interim analysis was conducted after the first seven participants to make adjustments to the sample size if required and therefore avoid patients undergoing DSE unnecessarily. Comparisons were made between HHC and control scans (study 1), or GLP-1 HHC and control HHC scans (study 2), with each patient acting as their own control. Continuous and discrete variables are expressed as mean ± standard deviation (SD) and compared by use of the paired Student’s *t* test (and repeated measures one-way ANOVA for regional function parameters) or the Wilcoxon signed-rank test where appropriate after testing for normality of distribution using the Shapiro–Wilk test. Categorical data are expressed as numbers (percentages) and compared by use of McNemar’s test. Two-tailed tests were used on all occasions, and a probability value of <0.05 was considered statistically significant. Intra- and inter-observer variations were calculated using the Bland–Altman method and expressed as the coefficient of variation (SD divided by the average value of the variable) ± the 95% limits of agreement.

## Results

### Study 1: effects of hyperglycemia on myocardial performance during dobutamine stress

#### Study population

Twelve patients were assigned to and completed study 1. The clinical characteristics of the subjects are shown in Table 1.

**Table 1:** Clinical data of participants

	Study 1 (n = 12)	Study 2 (n = 10)
Clinical characteristics		
Age (year)	64.8 ± 7.0	66.1 ± 4.6
Male sex	11 (92)	10 (100)
Type 2 diabetes	0	10 (100)
Weight (kg)	84 (10)	93 (14)
BSA (m^2^)	2.0 ± 0.1	2.2 ± 0.2
HOMA IR	1.3 ± 0.8	2.7 ± 1.3
HbA1c, (%)	–	7.0 ± 0.7
Coronary disease		
LAD stenosis	8 (67)	6 (60)
LCx stenosis	4 (33)	2 (20)
RCA stenosis	4 (33)	7 (70)
Single vessel CAD	8 (67)	7 (70)
Two vessel CAD	4 (33)	1 (10)
Three vessel CAD	0	2 (20)
Anti-anginal medications		
B-blocker	11 (92)	6 (60)
Calcium channel antagonist	1 (8)	7 (70)
Long-acting nitrate	8 (67)	3 (30)
Nicorandil	0	4 (40)
Oral hypoglycemic agents		
Metformin	–	4 (40)
Sulfonylurea	–	4 (40)
Thiazolidinedione	–	1 (10)

Data are presented as mean ± SD or n (%). *BSA* body surface area, *HOMA IR* homeostasis model assessment of insulin resistance, *LAD* left anterior descending artery, *LCx* left circumflex artery, *RCA* right coronary artery, *CAD* coronary artery disease.

#### Dobutamine stress echocardiography

The two DSE were conducted 8.5 ± 3.9 days apart. There were no differences in the rate-pressure products at baseline, peak stress or recovery between the two scans (Table 2). Target heart rate was achieved in 6 (50%) subjects in the control study and 7 (59%) in the HHC study. As per the protocol, the dobutamine infusions in the remaining subjects were terminated early due to the development of angina.

**Table 2:** Hemodynamic data during DSE scans in study 1

	Peak stress			Recovery		
	Control	HHC	*P*	Control	HHC	*P*
Heart rate (bpm)	129 ± 15	128 ± 17	0.87	81 ± 15	78 ± 11	0.32
Systolic blood pressure (mm Hg)	153 ± 29	156 ± 22	0.79	136 ± 27	137 ± 19	0.86
Diastolic blood pressure (mm Hg)	76 ± 13	78 ± 11	0.86	81 ± 11	82 ± 11	0.90
Rate-pressure product (mm Hg bpm)	19,540 ± 3,719	19,900 ± 3,948	0.95	11,010 ± 2,977	10,730 ± 2,209	0.68

#### Biochemistry

**Fig. 4:**
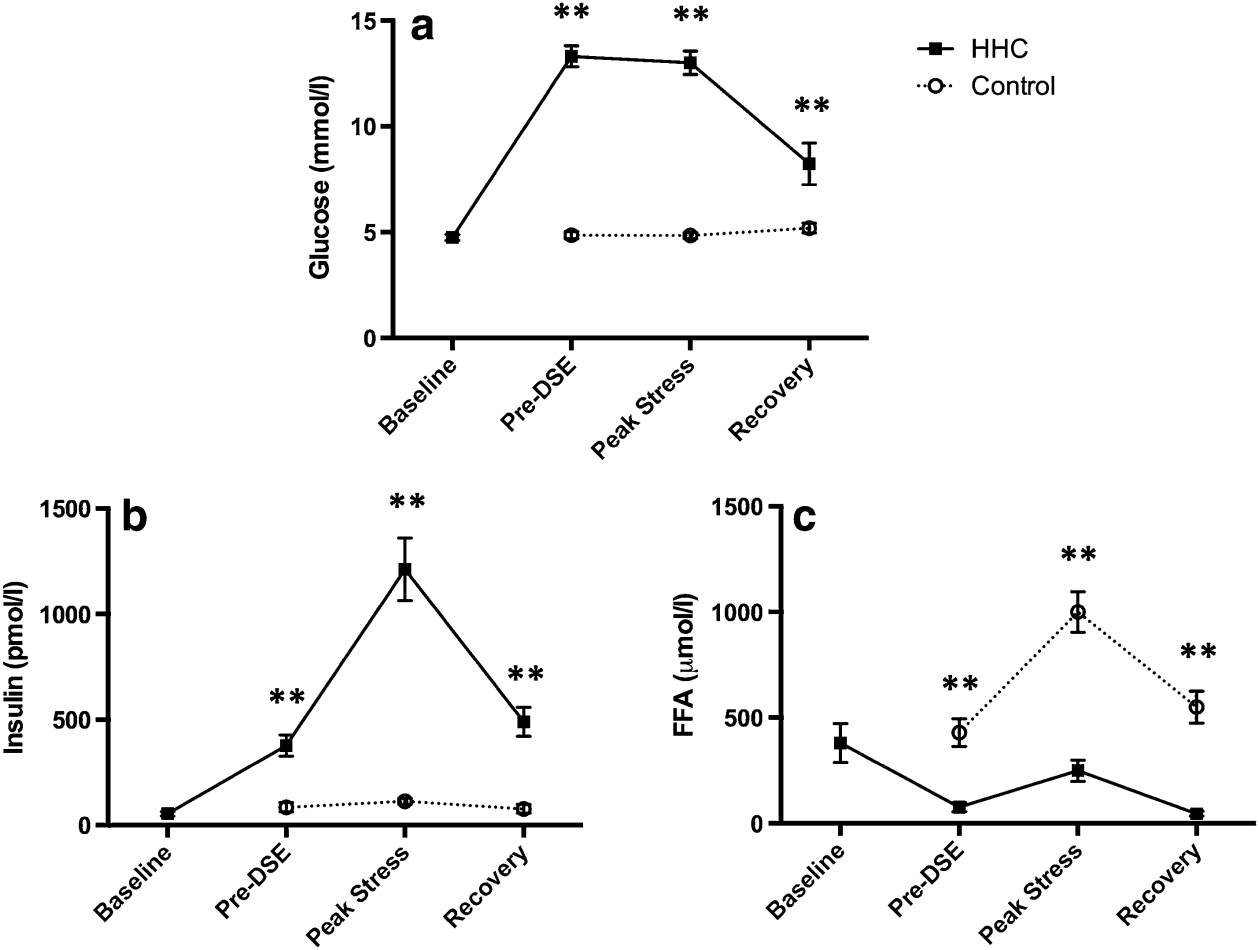
**a–c** Biochemical data (mean ± SEM) during the DSE scans in study 1 at baseline, pre-DSE, peak stress, and 30-min recovery. **p < 0.05.

At baseline, there were no differences in the plasma concentrations of glucose (4.8 ± 0.5 [HHC] versus 4.9 ± 0.6 mmol/l [control], p = 0.55), insulin (55 ± 39 [HHC] versus 86 ± 76 pmol/l [control], p = 0.18) or FFA (380 ± 320 [HHC] versus 429 ± 226umol/L [control], p = 0.36) between the HHC and control studies (Fig. 4). As intended, glucose concentrations at the steady state stage of the clamp (pre-DSE) were significantly higher than at baseline in the control study (13.3 ± 1.7 [HHC] versus 4.9 ± 0.6 mmol/l [control], p < 0.0001). This was associated with elevated insulin (378 ± 174 [HHC] versus 86 ± 76 pmol/l [control], p = 0.0007) and reduced FFA concentrations (77 ± 74 [HHC] versus 429 ± 226umol/l [control], p = 0.0003).

At peak stress, the metabolic differences between the two visits persisted, with higher glucose (13.0 ± 1.9 [HHC] versus 4.8 ± 0.5 mmol/l [control], p < 0.0001) and insulin (1,212 ± 514 [HHC] versus 114 ± 47 pmol/l [control], p = 0.01) concentrations, and reduced FFA levels (249 ± 175 [HHC] versus 1,001 ± 333 μmol/l [control], p < 0.0001), in the clamp study compared with control. In the recovery phase at 30 min after cessation of dobutamine (15 min after cessation of the glucose infusion), glucose (8.2 ± 3.4 [HHC] versus 5.2 ± 0.8 mmol/l [control], p = 0.005) and insulin (491 ± 240 [HHC] versus 79 ± 72 pmol/l [control], p = 0.02) concentrations remained higher and FFA concentrations lower (46 ± 40 [HHC] versus 550 ± 262 μmol/l [control], p < 0.0001) in the HHC study, although the magnitude of the differences was not as great.

#### Global LV function

**Fig. 5:**
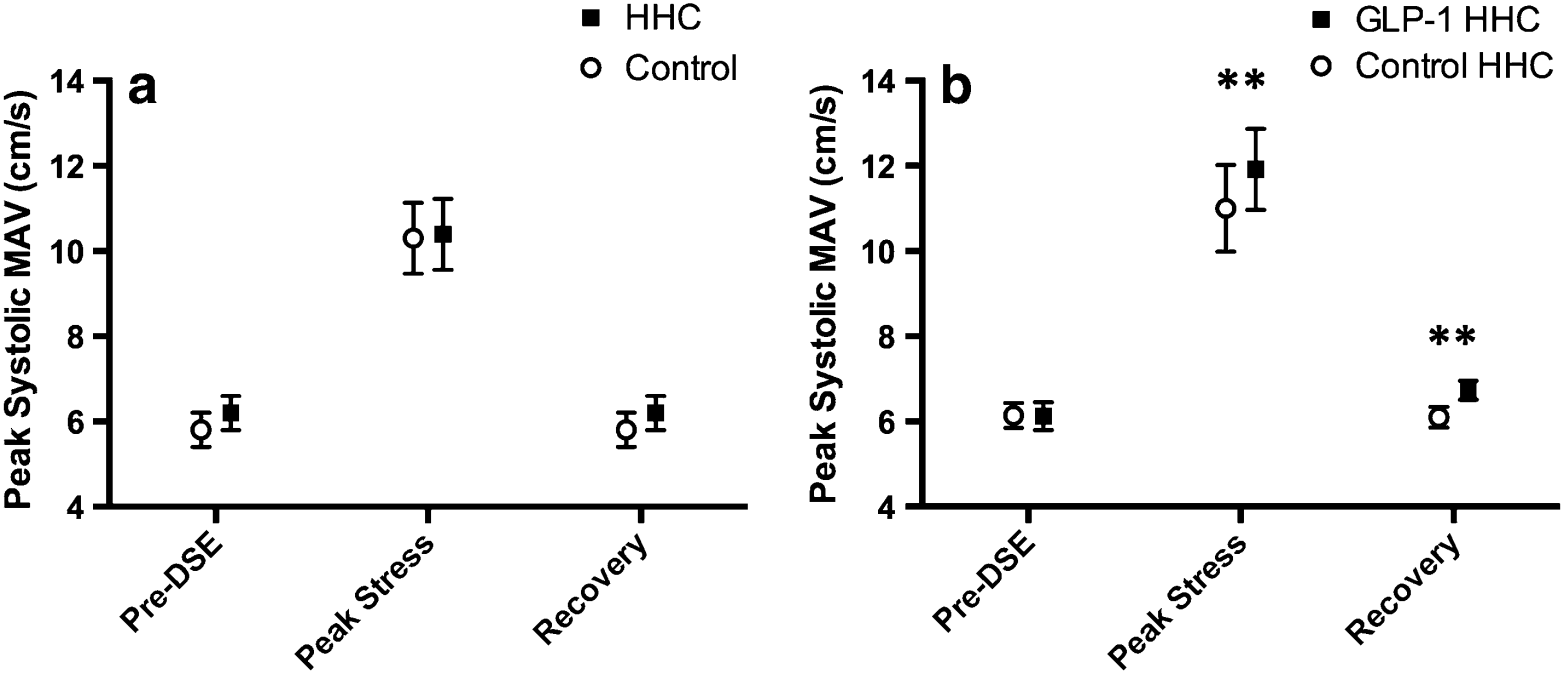
Global LV function assessed by peak systolic mitral annular velocity (mean ± SEM) at baseline, peak stress and 30-minute recovery in study 1 (**a**) and study 2 (**b**). **p < 0.05.

There were no differences in EF between the clamped and control studies at any stage (pre-DSE 66.3 ± 4.0 [HHC] versus 66.0 ± 4.7% [control], p = 0.76; peak stress 67.4 ± 11.0 [HHC] versus 69.1 ± 8.0% [control], p = 0.32; recovery 62.2 ± 4.0 [HHC] versus 63.5 ± 5.9% [control], p = 0.40). Assessment of global LV function by MASV confirmed these findings. The HHC was not observed to have any net effect on this parameter before, during or after dobutamine stress (pre-DSE 6.2 ± 1.4 [HHC] versus 5.8 ± 1.4 cm/s [control], p = 0.14; peak stress 10.4 ± 2.9 [HHC] versus 10.3 ± 2.9 cm/s [control], p = 0.68; recovery 6.2 ± 1.4 [HHC] versus 5.8 ± 1.4 cm/s [control], p = 0.12) (Fig. 5a).

#### Regional wall LV function

For the 12 paired non-apical segments, there were no differences in peak systolic tissue velocity, peak systolic strain or peak systolic strain rate between clamped and control studies either before the DSE or at peak stress. Similarly, at 30 min recovery, the HHC was not observed to have any effect on strain or strain rate, although it was associated with a small rise in tissue velocity (Table 3).

**Table 3:** Regional wall LV function in studies 1 and 2

Study 1	Pre-DSE			Peak stress			Recovery		
	Control	HHC	*P*	Control	HHC	*P*	Control	HHC	*P*
V_s_ (cm/s)	4.7 ± 1.7	4.8 ± 1.8	0.10	8.5 ± 3.3	8.6 ± 3.9	0.22	4.1 ± 1.6	4.6 ± 3.1	0.02
Strain (%)	−17.1 ± 6.9	−16.9 ± 7.7	0.46	−15.8 ± 7.0	−15.9 ± 6.9	0.62	−14.3 ± 6.9	−15.3 ± 7.0	0.06
Strain rate (s^−1^)	−1.1 ± 0.4	−1.1 ± 0.5	0.66	−1.8 ± 0.9	−1.8 ± 0.8	0.89	−1.0 ± 0.5	−1.0 ± 0.5	0.75

Study 2	Control	GLP-1	*P*	Control	GLP-1	*P*	Control	GLP-1	*P*
V_s_ (cm/s)	4.58 ± 1.62	4.56 ± 1.58	0.82	9.33 ± 3.25	9.78 ± 3.21	0.008	4.46 ± 1.48	5.01 ± 1.67	<0.0001
Strain (%)	−15.18 ± 5.94	−15.06 ± 5.05	0.77	−15.51 ± 5.39	−18.09 ± 6.58	<0.0001	−14.29 ± 5.35	−16.62 ± 5.71	<0.0001
Strain rate (s^−1^)	−1.04 ± 0.36	−1.06 ± 0.35	0.50	−2.02 ± 0.71	−2.30 ± 0.75	<0.0001	−0.96 ± 0.31	−1.18 ± 0.36	<0.0001

#### Ischemic versus non-ischemic segments

Regions subtended by an artery with a stenosis >70% on coronary angiography were defined as potentially subject to demand ischemia during DSE. The HHC was not observed to have any effect on parameters of regional wall function in either the ischemic or non-ischemic segments at any stage of the study, except for a small rise in tissue velocity in the non-ischemic segments (Table 4).

**Table 4:** Ischemic versus non-ischemic segments in study 1

Ischemic	Pre-DSE			Peak stress			Recovery		
	Control	HHC	*P*	Control	HHC	*P*	Control	HHC	*P*
V_s_ (cm/s)	4.54 ± 1.7	4.66 ± 1.7	0.14	8.54 ± 3.6	8.61 ± 4.7	0.44	3.94 ± 1.5	4.06 ± 1.9	0.12
Strain (%)	−16.73 ± 7.0	−16.68 ± 8.2	0.87	−14.93 ± 7.0	−15.21 ± 7.1	0.49	−14.33 ± 6.9	−14.72 ± 6.8	0.25
Strain rate (s^−1^)	−1.02 ± 0.4	−1.06 ± 0.5	0.44	−1.81 ± 1.0	−1.84 ± 0.8	0.36	−0.99 ± 0.6	−1.02 ± 0.5	0.32

Non-ischemic	Control	GLP-1	*P*	Control	GLP-1	*P*	Control	GLP-1	*P*
V_s_ (cm/s)	4.84 ± 1.8	4.95 ± 1.9	0.22	8.44 ± 3.0	8.56 ± 3.1	0.23	4.34 ± 1.7	5.06 ± 3.9	0.03
Strain (%)	−17.50 ± 6.8	−17.22 ± 7.2	0.32	−16.72 ± 7.1	−16.74 ± 6.8	0.77	−14.24 ± 7.0	−15.86 ± 7.3	0.06
Strain rate (s^−1^)	−1.12 ± 0.5	−1.10 ± 0.5	0.74	−1.74 ± 0.7	−1.78 ± 0.8	0.29	−1.04 ± 0.5	−1.07 ± 0.5	0.23

### Study 2: effects of GLP-1 (7-36) on myocardial performance during dobutamine stress in the setting of hyperglycemia

#### Study population

Interim analysis of the first seven patients had shown significant results, and therefore the number of subjects required was reduced. Ten patients were randomly assigned and completed study 2. The clinical characteristics of the subjects are shown in Table 1.

#### Dobutamine stress echocardiography

The two DSE were conducted 10.8 ± 7.2 days apart. There were no differences in the rate-pressure products at baseline, peak stress or recovery between the two scans (Table 5). Target heart rate was achieved in 6 (60%) subjects in the control HHC study and 5 (50%) in the GLP-1 HHC study. As per the protocol, the dobutamine infusions in the remaining subjects were terminated early due to the development of angina.

**Table 5:** Hemodynamic data during DSE scans in study 2

	Peak stress			Recovery		
	Control-HHC	GLP-1-HHC	*P*	Control-HHC	GLP-1-HHC	*P*
Heart rate (bpm)	126 ± 13	125 ± 9	0.83	71 ± 11	77 ± 9	0.14
Systolic blood pressure (mm Hg)	200 ± 25	206 ± 25	0.82	152 ± 22	146 ± 29	0.37
Diastolic blood pressure (mm Hg)	84 ± 24	81 ± 18	0.87	83 ± 14	84 ± 24	0.84
Rate-pressure product (mm Hg bpm)	25,177 ± 4,564	26,197 ± 3,883	0.92	10,733 ± 2,227	11,159 ± 2,788	0.54

#### Biochemistry

**Fig. 6:**
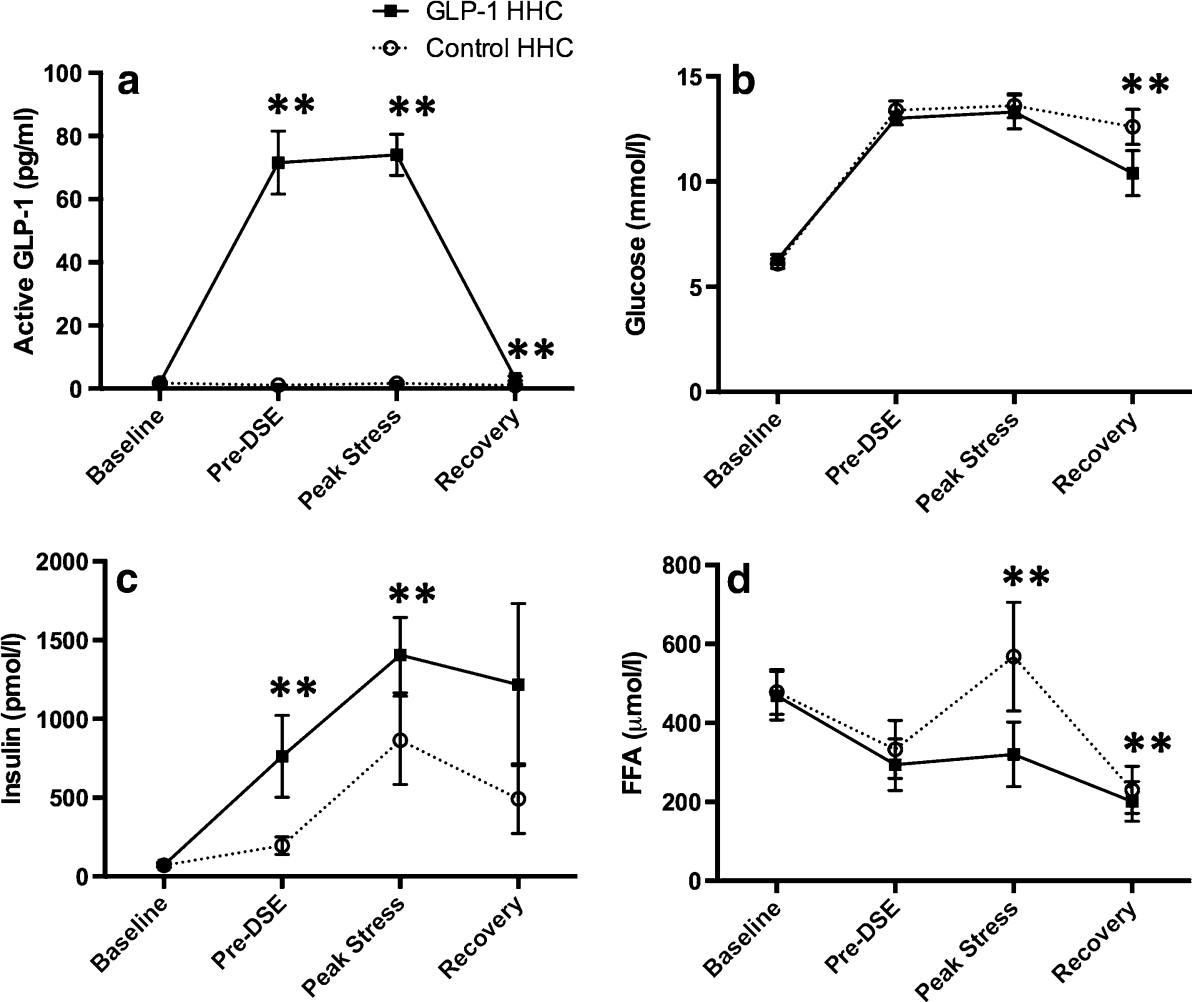
**a**–**d** Biochemical data (mean ± SEM) during the DSE scans in study 2 at baseline, pre-DSE, peak stress, and 30-min recovery. **p < 0.05.

At baseline, there were no differences in the plasma concentrations of glucose (6.3 ± 0.7 [GLP-1 HHC] versus 6.1 ± 0.7 mmol/l [control HHC], p = 0.36), insulin (73 ± 50 [GLP-1 HHC] versus 71 ± 36 pmol/l [control HHC], p = 0.76), FFA (469 ± 173 [GLP-1 HHC] versus 478 ± 160umol/L [control HHC], p = 0.78) or GLP-1(7-36) (1.8 ± 1.6 [GLP-1 HHC] versus 1.8 ± 1.3 pg/ml [control HHC], p = 0.92) between the GLP-1 and control studies (Fig. 6).

As intended, the GLP-1 infusion increased the plasma concentration of active GLP-1(7-36) amide approximately 50 times above the levels in control (pre-DSE 72 ± 28 [GLP-1 HHC] versus 1.1 ± 0.8 pg/ml [control HHC], p = 0.0001; peak stress 74 ± 17 [GLP-1 HHC] versus 1.7 ± 1.4 pg/ml [control HHC], p = 0.0001), and the HHC elevated plasma glucose concentrations during both visits to a similar level (pre DSE 13.0 ± 0.9 [GLP-1 HHC] versus 13.4 ± 1.3 mmol/l [control HHC], p = 0.20; peak stress 13.3 ± 2.4 [GLP-1 HHC] versus 13.6 ± 1.7 mmol/l [control HHC], p = 0.07). However, endogenous insulin production was greater (pre-DSE 763 ± 735 [GLP-1 HHC] versus 195 ± 157 pmol/l [control HHC], p = 0.03; peak stress 1,405 ± 634 [GLP-1 HHC] versus 864 ± 743 pmol/l [control HHC], p = 0.002) and FFA concentrations lower (pre-DSE 302 ± 198 [GLP-1 HHC] versus 359 ± 209umol/l [control HHC], p = 0.006; peak stress 321 ± 216 [GLP-1 HHC] versus 568 ± 364umol/l [control HHC], p = 0.03) after GLP-1 than in the control studies.

In the recovery phase at 30 min after cessation of dobutamine (15 min after cessation of the GLP-1 and glucose infusions), the GLP-1(7-36) amide concentration remained higher than in control (3.2 ± 1.9 [GLP-1 HHC] versus 1.0 ± 1.0 pg/ml [control HHC], p = 0.03) resulting in a lower glucose concentration (10.4 ± 3.2 [GLP-1 HHC] versus 12.6 ± 2.5 mmol/l [control HHC], p = 0.004). Insulin concentrations remained higher (1,218 ± 1,455 [GLP-1 HHC] versus 493 ± 586 pmol/l [control HHC], p = 0.05) and FFA concentrations lower (106 ± 50 [GLP-1 HHC] versus 230 ± 158μmol/l [control HHC], p = 0.02) in the GLP-1 study.

#### Global LV function

There was no difference in resting EF between the GLP-1 and control studies (pre-DSE 58.1 ± 4.5 [GLP-1 HHC] versus 59.3 ± 6.1% [control HHC], p = 0.41). At peak stress, there was a greater increase in LV function after GLP-1 infusion during hyperglycemia (77.5 ± 5.0 [GLP-1 HHC] versus 71.3 ± 4.3% [control HHC], p = 0.004), and this improved performance persisted into recovery (59.6 ± 3.6 [GLP-1 HHC] versus 50.1 ± 5.8% [control HHC], p < 0.0001). Assessment of global LV function by MASV confirmed these findings. LV function was similar before both scans (pre-DSE 6.1 ± 0.9 [GLP-1 HHC] versus 6.1 ± 0.8 cm/s [control HHC], p = 0.93). However, after GLP-1 infusion during hyperglycemia, myocardial performance was enhanced both at peak stress (11.9 ± 2.7 [GLP-1 HHC] versus 11.0 ± 2.9 cm/s [control HHC], p = 0.01) and at 30 min after dobutamine stress (6.7 ± 0.6 [GLP-1 HHC] versus 6.1 ± 0.7 cm/s [control HHC], p = 0.02) (Fig. 5b).

#### Regional wall LV function

For the 12 paired non-apical segments, there were no differences in peak systolic tissue velocity, peak systolic strain or peak systolic strain rate between the GLP-1 and control studies pre-DSE. However, at peak dobutamine stress, all three parameters of regional wall function were enhanced after GLP-1 infusion, and these improvements persisted into recovery (Tables 3, 6).

**Table 6:** Repeated measures analysis of regional wall function (control versus GLP-1 studies) at different time points in study 2

	Pre-DSE			Peak Stress			Recovery		
	Mean difference	95% CI of difference	*P*	Mean difference	95% CI of difference	*P*	Mean difference	95% CI of difference	*P*
V_s_ (cm/s)	−0.0002	−0.29 to 0.29	>0.99	−0.42	−0.80 to −0.04	0.02	−0.56	−0.86 to −0.26	<0.0001
Strain (%)	0.17	−1.18 to 1.52	>0.99	−2.94	−4.63 to −1.24	<0.0001	−2.25	−3.77 to −0.73	0.0006
Strain rate (s^−1^)	−0.01	−0.09 to 0.07	>0.99	−0.29	−0.49 to −0.08	0.001	−0.23	−0.32 to −0.13	<0.0001

#### Ischemic vs non-ischemic segments

GLP-1 infusion during hyperglycemia had a greater beneficial effect on ischemic than on non-ischemic segments (Table 7).

**Table 7:** Ischemic versus non-ischemic segments in study 2

Ischaemic	Pre-DSE			Peak stress			Recovery		
	Control	HHC	*P*	Control	HHC	*P*	Control	HHC	*P*
V_s_ (cm/s)	4.69 ± 1.53	4.66 ± 1.48	0.46	8.50 ± 3.07	8.70 ± 2.99	0.03	4.59 ± 1.53	4.90 ± 1.79	<0.0001
Strain (%)	−14.84 ± −5.51	−14.91 ± 4.39	0.82	−16.75 ± 5.26	−18.26 ± 7.38	<0.0001	−15.61 ± 5.47	−16.65 ± 5.72	0.006
Strain rate (s^−1^)	−0.99 ± 0.29	−1.00 ± 0.29	0.71	−2.02 ± 0.79	−2.17 ± 0.70	<0.0001	−1.10 ± 0.29	−1.21 ± 0.38	<0.0001

Non-ischaemic	Control	GLP-1	*P*	Control	GLP-1	*P*	Control	GLP-1	*P*
V_s_ (cm/s)	4.44 ± 1.73	4.46 ± 1.70	0.81	10.80 ± 3.05	11.06 ± 3.01	0.09	4.92 ± 1.41	5.15 ± 1.52	0.001
Strain (%)	−15.43 ± 6.41	−15.23 ± 5.75	0.48	−16.86 ± 5.59	−17.89 ± 5.57	0.007	−15.26 ± 5.27	−16.57 ± 5.79	0.0009
Strain rate (s^−1^)	−1.11 ± 0.41	−1.12 ± 0.39	0.55	−2.32 ± 0.70	−2.45 ± 0.79	0.006	−1.04 ± 0.32	−1.15 ± 0.35	<0.0001

### Reproducibility

We have previously assessed the reproducibility of the parameters presented in six randomly selected patients for the images recorded at rest, peak stress, and in recovery. The intra- and inter-observer variations for the tissue Doppler imaging parameters were, respectively, 6.12 ± 0.96% and 6.40 ± 0.99% for MASV, 7.22 ± 0.93% and 5.80 ± 0.76% for tissue velocity, 11.0 ± 3.56% and 12.6 ± 4.04% for strain, and 9.75 ± 0.29% and 11.6 ± 0.34% for strain rate. For LVEF, the intra-observer variation was 5.69 ± 7.00%, and the inter-observer variation was 6.59 ± 8.18%.

## Discussion

In these studies, we have demonstrated two key findings in patients with IHD: (1) HH has a neutral effect on LV function during dobutamine stress; and (2) in patients with T2DM, an intravenous infusion of GLP-1(7-36) amide at the time of hyperglycemia enhances myocardial performance during dobutamine stress.

### Hyperglycemia and the myocardium

It is well recognised that acute hyperglycemia may have a direct detrimental effect on ischemic myocardium. Although a large number of potential mechanisms have been postulated, those relevant to demand ischemia include increased oxidative stress and reduced nitric oxide production [23] leading to a reduction in flow-mediated, endothelium-dependent vasodilatation [24, 25], and increased production of endothelial vasoconstrictor prostanoids which impairs the acetylcholine-mediated vasodilator response [26]. In contrast, hyperinsulinemia is thought to have several beneficial effects on ischaemic myocardium, including a reduction in the circulating concentration of FFAs [27], and a subsequent shift towards greater myocardial glucose utilization, which improves overall myocardial oxygen efficiency [28].

In our study, the HHC stimulated endogenous insulin production and resulted in inhibition of lipolysis with an associated reduction in FFA concentration. At peak stress, the combination of pancreatic β1-stimulation from dobutamine and hyperglycemia led to a marked potentiation of insulin release, increasing further the differences between insulin and FFA. These metabolic differences persisted (but to a lesser degree) in the recovery phase at 30 min after cessation of dobutamine. However, despite these metabolic alterations, there was no significant net effect observed on either global or regional LV function during dobutamine stress or in recovery.

These findings are in contrast to the results of our previous work where we observed improved myocardial performance in patients with CAD undergoing DSE during the steady state stage of a HEC [17]. Whilst both clamp techniques would be expected to promote similar changes in myocardial metabolism (i.e. a transition from predominantly FFA oxidation to greater glucose utilization), HHC has been shown to reduce myocardial perfusion reserve when compared with HEC in both diabetic [29] and non-diabetic patients [30]. Therefore, the observed differences on LV function between the two clamp techniques might be explained by a counterbalance between the favourable effects of hyperinsulinemia on myocardial function and the inhibitory effects of hyperglycemia on coronary vasodilatation. In contrast to this hypothesis, Nielsen et al. found that short-term hyperglycemia (induced by discontinuation of insulin) in patients with T2DM increased LV contractility in patients with both normal and reduced EF. However, these results are not directly comparable to those of our study, which assessed non-insulin requiring diabetics using a different protocol to induce hyperglycemia (i.e. exogenous glucose clamp with subsequent large increase in insulin levels) [31].

### Insulin resistance and the myocardium

Insulin resistance refers to the reduced response of glucose uptake to the stimulatory effect of insulin. In the myocardium, insulin resistance suppresses oxidation of glucose, improves fatty acid metabolism and alters intracellular signalling, leading to numerous impairments of excitation–contraction coupling, less efficient energy production and increased susceptibility to ischemia/reperfusion injury [32]. Although these changes are common in patients with diabetes, they are frequently unrecognised. However, sensitive echocardiographic techniques such as TDI and SR imaging allow for earlier detection of asymptomatic LV dysfunction in these individuals. Compared with healthy age- and sex-matched controls, subjects with insulin resistance demonstrate a reduced increase in longitudinal SR values during DSE, suggesting reduced LV contractile reserve [33]. In patients with diabetes, longitudinal strain reserve (defined as the difference between global longitudinal strain before and after dipyridamole infusion) is increased compared to non-diabetics [34]. Furthermore, patients with T2DM and microvascular angina demonstrate reduced early diastolic peak velocities and peak systolic two-dimensional strain than healthy age- and sex-matched controls [35].

### GLP-1 and hyperglycemia

To the best of our knowledge, our study is the first to investigate the effect of GLP-1 modulation on LV function during myocardial ischemia in the setting of hyperglycemia in patients with T2DM and CAD. In study 2, there were no differences in GLP-1 levels or LV function between the two scans at baseline. Infusion of GLP-1 during HHC increased the plasma concentration of active GLP-1(7-36) amide approximately 50 times above the levels in control, resulting in increased insulin and suppressed FFA concentrations. Compared with control, GLP-1 was associated with improved parameters of both global and regional LV performance at peak stress and at 30 min recovery. These effects were predominantly driven by a reduction in contractile dysfunction in regions subject to demand ischemia. Glucose levels were similarly elevated in both scans, suggesting that the favorable cardiovascular effects were due to GLP-1 itself and not to euglycemia. Although insulin concentrations were higher during the GLP-1 scan, numerous studies have demonstrated that incretin-related cardioprotection occurs independent of an effect on insulin [14, 16, 36].

Over the last decade, there has been an increasing body of evidence highlighting the favorable cardiovascular properties of GLP-1 [37–39]. In animal studies that have assessed cardiac metabolic alterations, GLP-1 modulation was associated with increased myocardial glucose uptake [40, 41], reduced myocardial levels of lactate and pyruvate [42], and an increase in the relative oxidation of carbohydrate versus fat [41]. GLP-1 may therefore confer its cardioprotective effects by inducing a shift towards greater myocardial glucose utilization, an adaptive response which is known to be more oxygen efficient than fatty acid metabolism [43], but which is impaired in the context of insulin resistance. In keeping with this concept, we found that ischemic segments derive more benefit than non-ischemic segments.

Hyperglycaemia is known to be associated with an adverse prognosis in patients presenting with ACS [1]. However, attempts to improve outcomes with insulin-based strategies have proven to be ineffective. Intense insulin therapy (to achieve euglycemia) has been associated with increased mortality and hypoglycemia when compared with moderate glucose control [4]. Similarly, glucose-insulin-potassium infusions (which aim to achieve high insulin and circulating glucose levels in order to increase myocardial glucose uptake and inhibit the adverse effects of FFA) have also failed to demonstrate consistent clinical benefits [5]. The results of our study indicate that GLP-1 may offer an important therapeutic adjunct as a metabolic agent for cardioprotection in patients with T2DM.

## Limitations

This study has a number of important limitations. Firstly, we have used tissue Doppler-derived indices of myocardial deformation to assess regional LV function, which has a number of potential pitfalls compared with two-dimensional speckle tracking echocardiography (STE). These include a high dependence on a favorable angle of incidence during image acquisition, an inability to accurately assess apical segments, and potentially high inter-and intra-observer variations. However, since TDI allows for measurements of strain and strain rate with excellent temporal resolution, this technique may be advantageous to STE at times of higher heart rates (e.g. during dobutamine stress). Secondly, whilst all attempts were made to conduct the two DSE scans for each patient in identical fashion and to obtain the peak stress images at the same degree of dobutamine stress, we cannot exclude the possibility of a degree of variation in the response of individual patients to dobutamine on the two separate study days. Thirdly, the potential cardioprotective effects of GLP-1 have been assessed using echocardiographic (and not clinical) endpoints in only a small number of patients. Further work, in the form of adequately powered randomized controlled trials, is required to ascertain if these favorable cardiovascular effects at the time of demand ischemia translate into an improvement in clinical outcomes.

## Conclusions

In patients with obstructive CAD, hyperglycemic hyperinsulinemia has a neutral effect on LV function during dobutamine stress. However, an infusion of GLP-1(7-36) at the time of hyperglycemia protects the heart against demand ischemic LV systolic dysfunction in patients with T2DM and IHD.

## Abbreviations

ACS: acute coronary syndromes
FFA: free fatty acids
GLP-1: glucagon-like peptide-1
IHD: ischaemic heart disease
HEC: hyperinsulinemic euglycemic clamp
DSE: dobutamine stress echocardiography
CAD: coronary artery disease
LV: left ventricular
HH: hyperinsulinemic hyperglycemia
T2DM: type 2 diabetes mellitus
EF: ejection fraction
DPP4: dipeptidyl peptidase-4
HHC: hyperinsulinemic hyperglycemic clamp
TDI: tissue Doppler imaging
MASV: mitral annular systolic velocity
STE: speckle tracking echocardiography
